# Use of fermented Chinese medicine residues as a feed additive and effects on growth performance, meat quality, and intestinal health of broilers

**DOI:** 10.3389/fvets.2023.1157935

**Published:** 2023-03-28

**Authors:** Xinhong Zhou, Shiyi Li, Yilong Jiang, Jicheng Deng, Chuanpeng Yang, Lijuan Kang, Huaidan Zhang, Xianxin Chen

**Affiliations:** ^1^Leshan Academy of Agriculture Science, Leshan, Sichuan, China; ^2^College of Life Science and Engineering, Southwest University of Science and Technology, Mianyang, Sichuan, China

**Keywords:** broilers, fermented Chinese medicine residues, growth capacity, intestinal, meat quality

## Abstract

**Introduction:**

The purpose of this research was to investigate how dietary supplementation with fermented herbal residues (FCMR) affected birds' development capacity, quality of meat, gut barrier, and cecum microbiota.

**Methods:**

540 cyan-shank partridge birds aged 47 days were chosen and divided into two groups of six replicates each and 45 birds for each replicate. The control group (CON) received a basal diet, while the trial group decreased a basic diet containing 5% FCMR.

**Results and discussion:**

The findings revealed that the addition of FCMR decreased FCR and increased ADG in broilers (*P* < 0.05). Adding FCMR increased steaming loss in broiler chicken breasts (*p* < 0.05). Supplementation with FCMR significantly enhanced VH/CD and VH in the bird's intestine (jejunum, duodenum, and ileum) (*p* < 0.05). In addition, the addition of FCMR significantly down-regulated mRNA expression of INF-γ, IL-6, IL-1β, and TNF-α and up-regulated mRNA expression of ZO-1, Occludin, and Claudin (*P* < 0.05). Microbial 16S rDNA high-throughput sequencing study revealed that supplements with FCMR modified the cecum microbiota, and α-diversity analysis showed that supplementation with FCMR reduced the cecum bacterial abundance in broilers (*P* < 0.05). At the phylum level, the relative abundance of *Spirochaetota* increased considerably following FCMR supplementation (*P* < 0.05). The broiler cecum's close lot of *Prevotellaceae_UCG-001* (*P* < 0.05), *Desulfovibrio, Muribaculaceae*, and *Fusobacterium* (*p* < 0.05) reduced when FCMR was supplemented. Supplementation with FCMR can promote growth capacity and maintain intestinal health in birds by enhancing gut barrier function and modulating the inflammatory response and microbial composition.

## 1. Introduction

In recent years, antibiotic additives have played a significant role in preventing animal diseases and improving production performance (1). However, the massive application of antibiotics can lead to harmful strains of bacteria developing resistance, reduce the quality of livestock products, and even affect the ecological environment and threaten human health (2, 3). Therefore, the use of green and efficient methods to replace antibiotics has become popular research in the field of nutrition. Chinese medicine extract production process will produce many by-products; these by-products contain polysaccharides, alkaloids, amino acids, vitamins, and other active ingredients, which can improve animal performance by enhancing the body's immunity, and anti-inflammatory functions, as well as anti-bacterial, appetite, anti-stress, and other functions (4–7). If these herbal residues are discarded, it will result in a waste of resources and pollute the environment (8). However, due to the bitter taste of the herbal residues, feeding them directly to animals will affect their palatability and thus reduce their intake (9).

Biological fermentation technology combines fermentation engineering and enzyme engineering, which effectively reduces anti-nutritional factors in plant-based raw materials, increases active substances and bioactive ingredients in raw materials, improves feed palatability and processing performance, and makes it easy for animals to eat (10, 11). The microbial fermentation process in the cellulose cell wall, lignin and other substances is degraded, its active ingredients can be released, and the functional elements in the Chinese medicine residues are enzymatically dissolved into small molecules, which can enhance the effectiveness of the medicine (12–15). It was found that the inhibition of the growth of *Staphylococcus aureus, Salmonella cholerae*, and *Escherichia coli* may be increased by fermented herbs (9). Research has shown that fermented Chinese medicines can improve growth capacity, meat quality and immune performance in pigs (16), cattle (17), and ducks (18). Fermented Chinese medicines can regulate birds' antioxidant capacity, gut barriers, and microflora (19–21).

Gut health is strongly linked with the integrity of the gut barrier and the composition of gut microbiota (22, 23). An optimal intestinal wall prevents the invasion of harmful substances of exogenous origin and helps preserve gut balance and safeguard health (24). Therefore, this research was carried out to understand better how FCMR affects the growth, quality of meat, and intestinal health of chicks. To provide data support for the rational use of FCMR on broiler chickens and provide a scientific basis for chickens' healthy breeding.

## 2. Materials and methods

### 2.1. Animals

All broilers are sourced from a local commercial farm (Leshan, Sichuan, China) and broiler breeding trials are conducted at this commercial farm (Lihui Family Farm). At the beginning of the trial, we weighed all chickens after 12 h of starvation and selected 540 47-day-old male cyan-shank partridge chickens of similar body weight randomly divided into two groups, with six parallel pens (45 birds per pen, 120 cm−60 cm−50 cm). The rearing period continued for 42 days. The control group (CON) received a basal diet, while the trial group decreased an essential diet containing 5% FCMR. Chickens were allowed to feed and drink freely every day, and the daily amount of remaining feed was collected to calculate the daily feed intake. The chicken coop has a temperature of 18–25°C, a humidity of 45%−55%, and receives natural light every day. All chickens in each replicate were weighed after 12 h of starvation on day 42, and average daily feed intake (ADFI), average daily gain (ADG), and feed conversion ratio (FCR) were calculated based on feed consumption.

### 2.2. Diets and feeding

The basal feed formulas and nutrients for this experiment are shown in Table 1, and the FCMR formulas and nutrients are shown in Table 2. The Chinese medicine residues in FCMR are the by-product of the extraction or decoction of Chinese medicines with the ingredients isatis root, spatholobus stem, radix bupleuri, Poria Cocos, and Sedum sarmentosum Bunge. The FCMR is made using a synergistic fermentation method of probiotics and enzymes. Briefly, Chinese medicine residues and other ingredients (Table 2) are crushed and mixed thoroughly, then put into fermentation bags with a one-way breathing valve, fermented at 36°C for 3 days and ready for use. The probiotics used for fermentation contain *Lactobacillus plantarum, Bacillus subtilis*, and *Saccharomyces cerevisiae*. The enzymes contained: cellulase, xylanase, β-glucanase, and β-mannanase.

**Table 1:** The ingredient composition of the basal diet and nutrient levels.

**Items^a^**	**Content (%)**	**Nutritional level^b^**	**Content (%)**
Corn	47.4	Metabolic energy (MJ/kg)	12.83
Wheat middlings	14	Crude protein	19.5
Soybean meal	7	Crude Ash	9.08
Cottonseed meal	5	Moisture	11.25
Canola meal	3	Ca	0.73
Corn protein powder	4.9	P	0.37
Corn bran residue	8	Lysine	0.99
Chicken Powder	4	Methionine	0.44
Pork oil	3.35		
CaHPO_4_	1.15		
Stone powder	0.4		
NaCl	0.32		
Lysine	0.48		
Premixes	1		
Total	100		

a Premix is provided per kg: V_A_ 8,000 IU, V_D_ 1,700 IU, V_E_ 13 IU, V_K_ 0.9 mg, VB_1_ 1 mg, VB_2_ 3 mg, VB_6_ 1.3 mg, VB_12_ 0.01 mg; Pantothenic acid 12 mg, Niacin 16 mg, Folic acid 3 mg, Fe 55 mg, Cu 25 mg, Zn 35 mg, Mn 60 mg, Se 0.3 mg, I 0.8 mg.

b Nutrient levels are measured except for metabolic energy.

**Table 2:** The ingredient composition of FCMR and nutrient levels.

**Items**	**Content (%)**	**Nutritional level^b^**	**Content (%)**
Corn	14.76	Metabolic energy (MJ/kg)	8.2
Soybean meal	17.63	Crude protein	24.02
Corn bran residue	15.08	Crude Ash	8.80
FCRM	20.6	Moisture	29.53
Bran	3	Ca	0.82
Rice husk powder	3	P	0.5
H_2_O	23.71	Lysine	0.74
Stone powder	0.62	Methionine	0.64
Premixes^a^	0.25		
CaHPO_4_	0.59		
NaCl	0.36		
NaHCO_3_	0.1		
Methionine	0.1		
Compound bacteria	0.1		
Enzyme preparation	0.1		
Total	100		

a Premix is provided per kg: V_A_ 8,000 IU, V_D_ 1,700 IU, V_E_ 13 IU, V_K_ 0.9 mg, VB_1_ 1 mg, VB_2_ 3 mg, VB_6_ 1.3 mg, VB_12_ 0.01 mg; Pantothenic acid 12 mg, Niacin 16 mg, Folic acid 3 mg, Fe 55 mg, Cu 25 mg, Zn 35 mg, Mn 60 mg, Se 0.3 mg, I 0.8 mg.

b Nutrient levels are measured except for metabolic energy.

### 2.3. Sample collection

At the end of the experiment, broilers were fasted for 12 h before weighing, and one was randomly selected from each replicate, for a total of 12 birds. The broilers were euthanized by cervical dislocation and necropsied immediately. The pectoralis major muscle from the same side was then taken for meat quality analysis, and a portion was stored in a refrigerator at 4°C for meat quality trait analysis and another portion was stored at −80°C for amino acid composition analysis of the pectoralis muscle. After the empty intestine was gently washed off the contents with saline, about 2 cm of the intestine was taken and placed in 4% paraformaldehyde solution for paraffin sectioning, and then the remaining portion of the empty intestine was placed in a −80 refrigerator for gene expression measurement. Cecal contents were also collected for 16S rDNA gene sequencing.

### 2.4. Meat quality analysis

#### 2.4.1. Meat quality traits

Using a piercing electrode coupled to a mobile pH-meter (Testo 205, Germany) into the quadriceps forms and pectoralis major muscle, the pH of the pectoral muscle was determined at around 45 min and 24 h postoperatively. Before use, the pH meter was validated using standard phosphate buffer at pH 4 and 7.

Using a portable color scanner, the color evaluations of the chicken meat (a^*^ = redness, b^*^ = yellowness, and L^*^ = brightness) were taken (3NH Technologies, China). The measurements are collected from the sample's inner side, representing the flesh's entire surface. A white tile (a^*^ 16.22, b^*^ 6.23, and L^*^ 3.76) was chosen as a reference.

Approximately 2 × 2 × 2 cm of chicken breasts were used to measure the muscle's drip loss, steaming loss, and freezing loss (25). Drip loss: the samples were measured and recorded as *W*_1_, then the powers were tied with thin string, put in an air-filled poly pouch, and hung at 4°C for 24 h, then weighed and recorded as *W*_2_. Steaming loss: the samples were measured and recorded as *W*_3_, then put in a polyethylene film, heated in a water shower at 70°C for 30 min, and weighed as *W*_4_. Freezing loss: the muscle samples were weighed and recorded as *W*_5_, placed in a plastic bag, and placed in a −20°C refrigerator for 24 h, thawed and weighed as *W*_6_.

- Drip loss = (*W*_1_ – *W*_2_)/*W*_1_ × 100 %
- Steaming loss = (*W*_3_ – *W*_4_)/*W*_3_ × 100 %
- Freezing loss = (*W*_5_ – *W*_6_)/*W*_5_ × 100 %.

#### 2.4.2. Determination of amino acids

The preserved muscle samples were taken, and the amino acid content was determined using an amino acid auto-analyzer. Briefly, a suitable amount of well-mixed piece (about 1 g) was weighed, and the sample was hydrolyzed in a vacuum-sealed glass tube with 6 mol/L HCl for 22 h at 110 ± 1°C. The solution was filtered using a 0.45 μm membrane filter into an autosampler vial, and then analyzed by L-8900 amino acid analyzer (Hitachi LA8080).

### 2.5. Jejunal histomorphology

According to our previous method (26), ~2 cm of duodenum, jejunum, and ileum were removed and secured with 4% paraformaldehyde. The intestinal tissues were dehydrated using ethanol, then cleaned with xylene, followed by wax immersion, embedding, sectioning (μm), and hematoxylin-eosin (HE) staining, and observed under a microscope for photographs. The VH and CD of each intestinal segment were measured by Image-Pro Plus, software and the VH/CD values were calculated.

### 2.6. Jejunal gene expression

Intestinal tissues stored at −80°C were placed in RNAase-free centrifuge tubes and ground using a microtissue homogenizer. Total RNA was extracted from the intestinal tissues by the Trizol method (TaKaRa, Japan). The integrity and concentration of RNA were examined by 1% agarose polyacrylamide gel electrophoresis and micro ultraviolet spectrophotometer. The first-strand cDNA was synthesized using the kit (TaKaRa, Japan) according to the instructions. Protocol for reverse transcription: 37°C for 15 min; 85°C for 5 s. The PCR amplification reactions were carried out with the CFX96 real-time quantitative fluorescence PCR assay system (Bio-Rad, USA) according to the kit's instructions (TaKaRa). The total amount of the real-time qPCR reaction system was 20 μl. Thermocycling amplified procedures included denaturing for 15 s at 95°C, heating for 15 s at 56°C, extending for 30 s at 72°C, and continuing for 40 cycles. Using GADPH as the inner reference gene, the mRNA expression level of targeted genes was obtained by the 2^−ΔΔCt^ approach (27). The Table 3 showed primers sequences used in this study.

**Table 3:** Primer sequences were used for the real-time PCR analysis.

**Genes**	**Sequence 5′-3′**	**GenBank number**
GAPDH	F: GGAAAGTCATCCCTGAGCTGAAT	NM_204305.1
	R: GGCAGGTCAGGTCAACAACA	
ZO-1	F: AATACCTGACTGTCTTGCAG	XM_015278975.1
	R: TAAAGAAGGCTTTCCCTGAC	
Occludin	F: GCAGATGTCCAGCGGTTACTAC	NM_205128.1
	R: CGAAGAAGCAGATGAGGCAGAG	
Claudin	F: CATACTCCTGGGTCTGGTTGGT	NM_001013611.2
	R: GACAGCCATCCGCATCTTCT	
IL-1β	F: ACTGGGCATCAAGGGCTA	XM_015297469
	R: GGTAGAAGATGAAGCGGGTC	
IL-6	F: GAAATCCCTCCTCGCCAATCT	XM_0152812832
	R: CCTCACGGTCTTCTCCATAAACG	
TNF-α	F: TGTGTATGTGCAGCAACCCGTAGT	NM_204267
	R: GGCATTGCAATTTGGACAGAAGT	
IFN-γ	F: AAAGCCGCACATCAAACACA	NM_205128.1
	R: GCCATCAGGAAGGTTGTTTTTC	

### 2.7. 16S rDNA-based sequencing analysis

The MagPure Soil DNA LQ kit was used to extract microbial DNA from 200 mg cecal content samples obtained from all groups. A Thermo Fisher Scientific NanoDrop 2000 spectrometer and electrophoresis on agarose gels were used to assess the DNA's concentration and integrity. TksGflflex DNA polymerase (Takara, R060B) and the universal primer pairs 343F (5′-TACGGRAGGCAGCAG-3′) and 798R (5′-AGGGTATCTAATCCT-3′) were used. Afterward, PCR amplification of the high variant region V3–V4 in the 16S rDNA gene of bacteria was performed in 30 ml of the reaction mixture. Two paired-end read cycles of 250 bases each were used for reading an Illumina NovaSeq6000. The sequencing and profiling of 16S rDNA amplicons were performed by OE (Shanghai, China).

### 2.8. Statistical analysis

The experimental results were expressed as mean ± SD, and independent samples *t*-test in SPSS 23.0 software was applied to analyze the significance of differences in growth performance, meat quality, intestinal morphology, and relative levels of gene expression, with *p* < 0.05 indicating significant differences.

The Quantitative Insights Into Microbial Ecology (QIIME, version 1.17) was used to demultiplex and quality-filter raw pair-end sequences for microbiota characterization. For rarefaction curves and α-diversity analysis were calculated using QIIME. β-diversity was estimated using principal coordinate analysis (PCoA). R software's “vegan” and “ggplot2” packages were used to plot the findings (Version 3.4.4). The significance of microbial community differences among groups was assessed using ANOSIM with R package “vegan” Before Linear discriminant analysis (LDA) combined effect size (LEfSe) estimate the impact of the abundance of bacteria on the difference effect of bacteria among groups (LDA > 3, *p* < 0.05), non-parametric factorial Kruskal–Wallis sum-rank test was employed to explore the differences in the relative abundances of bacteria among groups. Correlations were analyzed using spearman correlation with the pheatmap package (*p* < 0.05).

## 3. Results

### 3.1. Growth performance

The effects of FCHM on the growth performance of broilers are presented in Table 4. Supplementation with FCHM significantly enhanced FW and ADG in broilers (*p* < 0.05). Supplementation with FCHM significantly reduced FCR in broilers (*p* < 0.05). Supplementation with FCHM reduced ADFI and mortality in broilers, the differences were not statistically significant (*p* > 0.05).

**Table 4:** The effect of FCHM on growth performance of broiler chickens.

**Items**	**CON**	**FCHM**	***p*-value**
IW (kg)	1.48 ± 0.06	1.46 ± 0.06	0.688
FW (kg)	2.92 ± 0.15^*^	3.11 ± 0.84^*^	0.21
ADFI (g)	157.70 ± 1.56	148.61 ± 6.20	0.053
ADG (g)	34.40 ± 3.86^*^	39.35 ± 3.09^*^	0.034
FCR (g/g)	4.60 ± 0.48^*^	3.80 ± 0.39^*^	0.010
Mortality (%)	9.69 ± 4.84	7.01 ± 3.24	0.287

* Represents a remarkable difference among the two groups (n = 6, *p* < 0.05).

CON, control group, fed basic diet; FCHM, 5% FCHM replacement group.

### 3.2. Meat quality traits

Table 5 summarizes the effect of FCHM supplementation on meat quality traits of broilers. Supplementation with FCHM did not have a significant effect on the meat color and pH of broiler breast meat (*p* > 0.05). However, FCHM supplementation significantly reduced drip loss (*p* > 0.05) and steaming loss (*p* < 0.05) in broiler chicken breasts.

**Table 5:** Effects of FCHM on meat quality of broilers.

**Items^a^**	**CON**	**FCHM**	***p*-value**
a^*^	9.45 ± 1.64	8.77 ± 1.08	0.416
b^*^	13.09 ± 1.98	12.64 ± 1.41	0.663
L^*^	54.65 ± 12.96	47.93 ± 6.48	0.292
pH_45min_	5.61 ± 0.22	6.07 ± 0.30	0.170
pH_24h_	5.54 ± 0.0.10	5.31 ± 0.53	0.168
Drip loss	0.17 ± 0.14	0.07 ± 0.08	0.106
Steaming loss	0.33 ± 0.03^*^	0.28 ± 0.02^*^	0.011
Freezing loss	0.062 ± 0.027	0.061 ± 0.03	0.924

* Represents a remarkable difference among the two groups (n = 6, *p* < 0.05).

a CON, control group, fed basic diet; FCHM, 5% FCHM replacement group; a^*^, redness; L^*^, brightness; b^*^, yellowness.

### 3.3. Amino acids

The effect of the dietary addition of FCHM on the amino acid profile were presented in Table 6. The addition of FCHM decreased the level of serine (*p* < 0.05), which is a non-essential amino acid. Supplementation with FCHM had no significant effects on broiler muscle TAA, EAA, NEAA and DAA (*p* > 0.05).

**Table 6:** The effects of FCHM on amino acid profiles in broiler breast muscle.

**Items^a^**	**CON**	**FCHM**	***p*-value**
Aspartic acid	1.90 ± 0.07	1.84 ± 0.07	0.219
Threonine	0.91 ± 0.03	0.88 ± 0.03	0.190
Serine	0.76 ± 0.03^*^	0.69 ± 0.03^*^	0.007
Glutamic acid	2.91 ± 0.13	2.81 ± 0.13	0.254
Glycine	0.96 ± 0.22	0.82 ± 0.04	0.202
Alanine	1.19 ± 0.06	1.12 ± 0.44	0.096
Cystine	0.15 ± 0.01	0.13 ± 0.03	0.253
Valine	0.98 ± 0.04	1.00 ± 0.30	0.364
Methionine	0.41 ± 0.06	0.45 ± 0.09	0.388
Isoleucine	0.91 ± 0.05	0.94 ± 0.04	0.363
Leucine	1.67 ± 0.07	1.62 ± 0.06	0.779
Tyrosine	0.65 ± 0.03	0.65 ± 0.03	0.879
Phenylalanine	0.81 ± 0.03	0.79 ± 0.03	0.337
Lysine	1.81 ± 0.09	1.80 ± 0.06	0.902
Histidine	0.68 ± 0.08	0.71 ± 0.05	0.469
Arginine	1.30 ± 0.05	1.25 ± 0.04	0.171
Proline	0.71 ± 0.10	0.69 ± 0.01	0.635
TAA	18.68 ± 0.63	18.23 ± 0.69	0.309
EAA	9.41 ± 0.33	9.27 ± 0.35	0.536
NEAA	10.37 ± 0.39	10.07 ± 0.39	0.264
DAA	6.96 ± 0.28	6.60 ± 0.27	0.074

* Represents a remarkable difference among the two groups (n = 6, *p* < 0.05).

a CON, control group, fed basic diet; FCHM, 5% FCHM replacement group; ^a^TAA, total amino acids; EAA, essential amino acids; NEAA, non-essential amino acids; DAA, delicious amino acids.

### 3.4. Intestinal histomorphology

**Figure 1:**
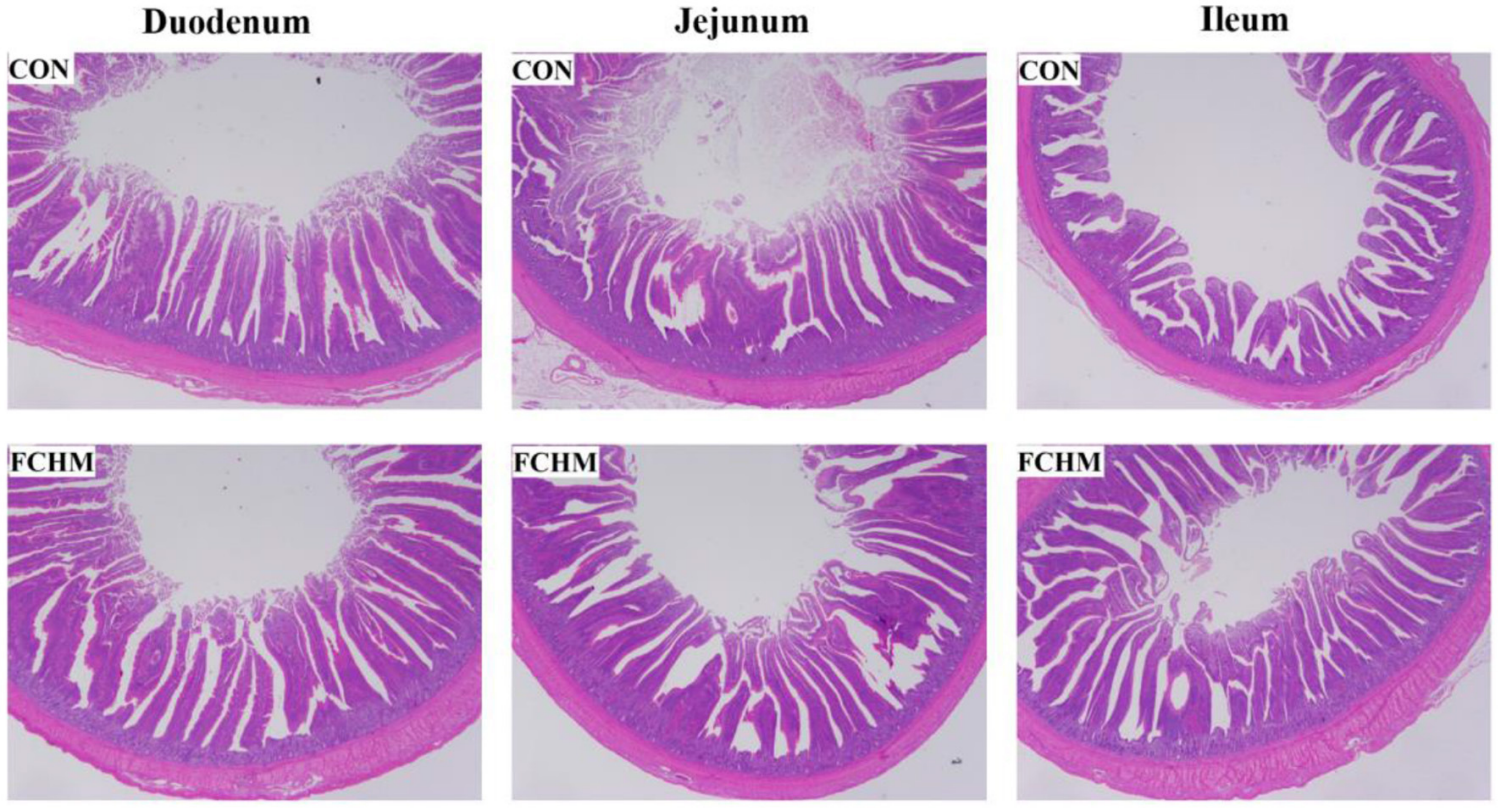
The morphologic structures of the gut (duodenum, jejunum, and ileum) of broiler chickens on day 42. The photos were viewed under 20× magnification. CON, control group, fed essential diet; FCHM, 5% FCHM replacement group.

Light microscopic photos of the gut morphology are presented in Figure 1. It can see that supplementation with FCHM improved the height of the villi in the small broiler intestine. The effects of FCHM on broiler intestinal villi are shown in Table 7. Supplementation with RCHM significantly enhanced VH/CD and VH in the broiler intestine (duodenum, jejunum, and ileum; *p* < *0.05*). The addition of FCHM significantly decreased CD in the duodenum and jejunum of broiler chickens (*p* > 0.05).

**Table 7:** Effects of FCHM supplementation on gut morphology of broilers.

**Items^a^**	**CON**	**FCHM**	***p*-value**
**Duodenum**			
VH (μm)	792.28 ± 38.73^*^	860.73 ± 29.81^*^	0.006
CD (μm)	115.76 ± 6.70^*^	96.62 ± 11.01^*^	0.005
VH/CD	6.87 ± 0.66^*^	9.03 ± 1.26^*^	0.004
**Jejunum**			
VH (μm)	769.52 ± 54.76^*^	859.31 ± 24.18^*^	0.008
CD (μm)	145.58 ± 15.76^*^	105.19 ± 5.34^*^	0.001
VH/CD	5.34 ± 0.70^*^	8.18 ± 0.34^*^	<0.001
**Ileum**			
VH (μm)	510.72 ± 18.59^*^	706.09 ± 13.97^*^	<0.001
CD (μm)	60.80 ± 7.70	66.95 ± 9.26	0.24
VH/CD	8.48 ± 0.79^*^	10.71 ± 1.41^*^	0.007

* Represents a remarkable difference among the two groups (n = 6, *p* < 0.05).

a CON, control group, fed basic diet; FCHM, 5% FCHM replacement group; VH, villous height; CD, crypt depth; VH/CD, villous height/ crypt depth; μm, micron.

### 3.5. The mRNA expression levels of tight junction protein and immunomodulatory genes

**Figure 2:**
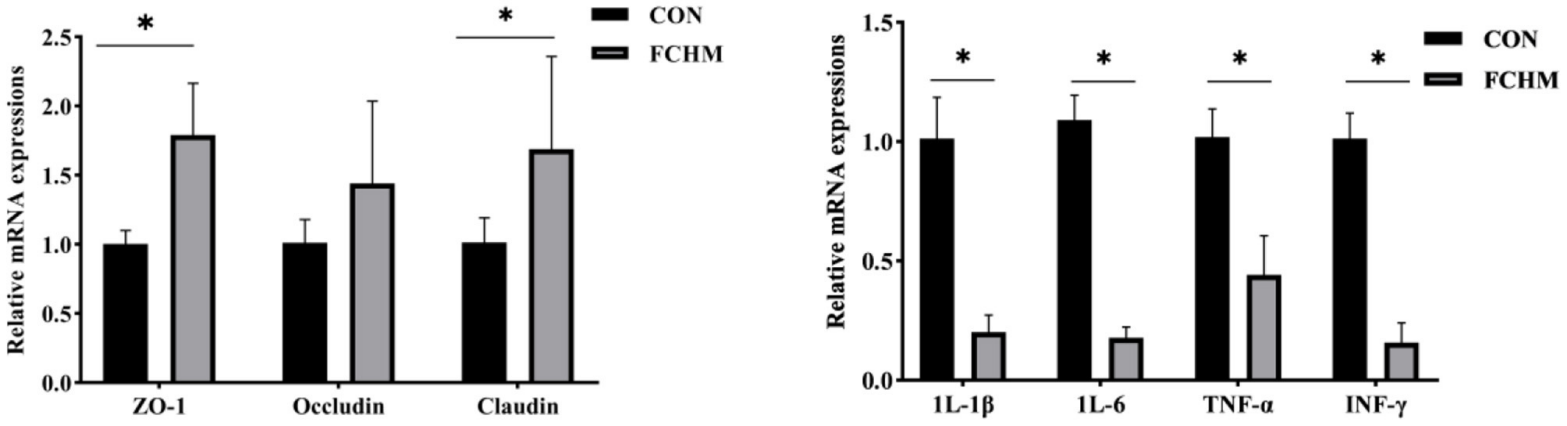
The mRNA expression level of tight junction protein and immunomodulatory genes in broilers jejunum. *Represents a remarkable difference among the two groups (*n* = 6, *p* < 0.05); ZO-1, zonula occluden-1; IL-1β, interleukin-1β; IL-6, interleukin-6; TNF-α, tumor necrosis factor-α; INF-γ, interferon-γ.

The expression levels of mRNA for tight junction proteins and immunoregulatory genes in broiler jejunum are shown in Figure 2. Supplementation with FCHM enhanced the expression of relative mRNA in broiler jejunum for tight junction proteins (ZO-1, Occludin, and Claudin; *p* < 0.05). Supplementation of FCHM significantly decreased the relative mRNA expression of immune genes (1L-1β, 1L-6, TNF-α, and INF-γ) in broiler jejunum (*p* < 0.05).

### 3.6. Cecal microbiota analysis by 16S rDNA

**Figure 3:**
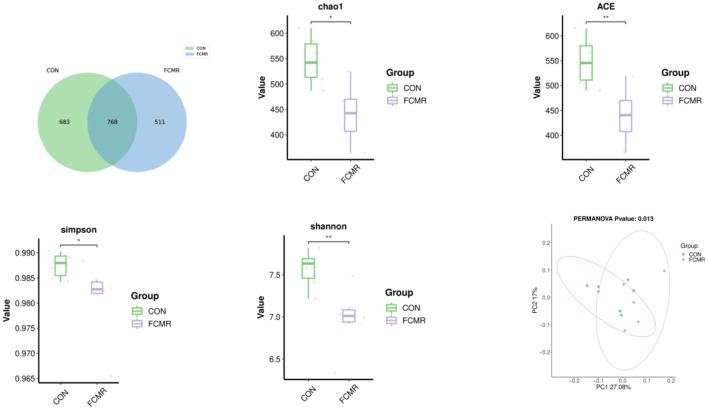
The effects of FCMR addition to the diet on the Venn diagram, α-diversity, and β-diversity of broiler cecum microorganisms. Principal coordinate analysis (PCoA) calculated based on weighted UniFrac distances from the ASV abundance matrix; *indicates that there is a considerable difference between the two groups (*p < 0.05, **p < 0.01).

There were 1,962 ASVs detected in broiler cecum feces, and 768 ASVs were shared between the two groups, with the CON and FCMR groups specific for ASV numbers 683 and 511 (Figure 3). Supplementation with FCHM significantly reduced the indices of cecum Chao1, Simpson (*p* < 0.05), and ACE, Shannon indices (*p* < 0.01). Significant differences between two groups of cecum microbiota based on PCoA (*p* < 0.05).

**Figure 4:**
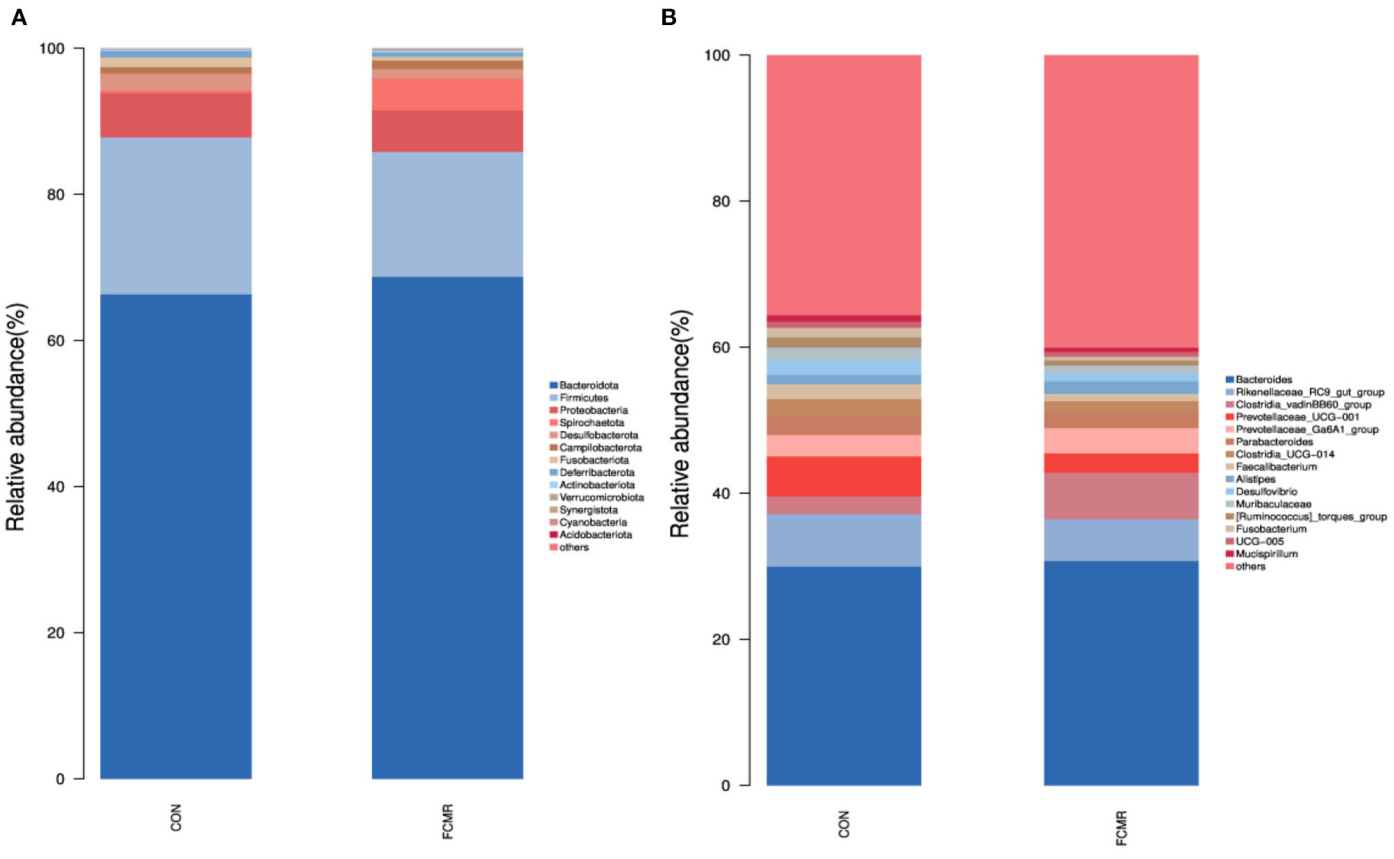
The microbiota composition and identification of differential species in the cecum of birds. **(A, B)** The phylum and genus level compositions of the bacterial communities, respectively.

At the phylum level, *Bacteroidota* and *Firmicutes* dominated both groupings (Figure 4A). Adding FCMR decreased the relative abundance of *Proteobacteria* at the gate level (6.05 vs. 5.67%). After supplementation with FCMR compared to the control group, the close lot of *Spirochaetota* increased considerably (0.36 vs. 4.36%, *p* < 0.05). At the genus level, we identified the top 15 wealthiest genera (Figure 4B). The dominant species between the two groups included *Bacteroides, Rikenellaceae_RC9_gut_group, Clostridia_vadinBB60_group, Prevotellaceae_UCG-001, Prevotellaceae_Ga6A1_group*. *Parabacteroides, Clostridia_UCG-014, Faecalibacterium*. Adding FCMR significantly enhanced the relative abundance of *Clostridia_vadinBB60_group* in broiler cecum (2.45 vs. 6.40%, *p* < 0.05). Adding FCMR significantly reduced the close lot of Prevotellaceae_UCG-001 (5.47 vs. 2.63%, *p* < 0.05). Feeding FCMR reduced the relative abundance of *Desulfovibrio, Muribaculaceae*, and *Fusobacterium* in the broiler cecum (*p* > 0.05).

**Figure 5:**
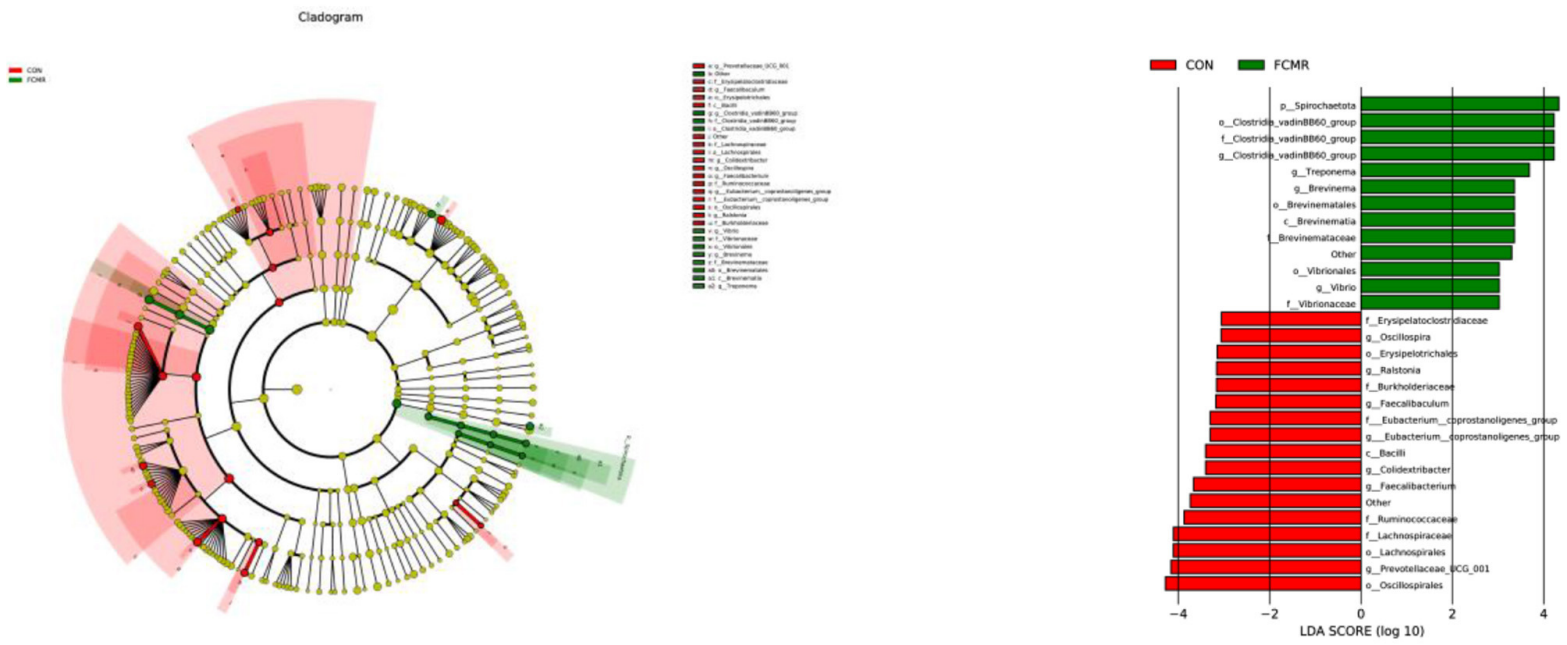
The microbiomes of the two groups were described using LEfSe and LDA analyses based on ASVs disparities in the (log LDA >3.0; *n* = 6).

Moreover, we explored species with marked differences using the LEFSE approach and indicated them with cladograms and LDA scores (Figure 5). At the phylum level, we found that *Spirochaetota* is a potential biomarker in the FCMR group. At the Class level, Bacilli was enriched in the CON group, and *Brevinematia* was increased in the FCMR group. Lachnospirales, Erysipelotrichales, and Oscillospirales were enriched in the CON group, Clostridia_vadinBB60_group, at the Order level *Brevinematales*, and *Vibrionales* were improved in the FCMR group. At the Family level, *Lachnospiraceae, Ruminococcaceae, Faecalibacterium*, and *Erysipelatoclostridiaceae* are potential biomarkers in the CON group, *Clostridia_vadinBB60_group Brevinemataceae* and *Vibrionaceae* were potential biomarkers in the FCMR group. Prevotellaceae_UCG_001, Faecalibacterium, Colidextribacter, Faecalibaculum, Ralstonia and Oscillospira we, re potential at the Genus level biomarkers in the CON group, *Clostridia _vadinBB60_group, Treponema, Brevinema* and *Vibrio* were potential biomarkers in the FCMR group.

## 4. Discussion

Microbial fermentation can boost a feed's nutritional content and make it more palatable, increase beneficial metabolites, and increase the utilization efficiency of feed as well as the growth performance of livestock and poultry (28, 29). We found that supplementation with FCMR increased ADG and decreased FCR in broilers, this may be due to the fact that supplementation with FCMR can better facilitate the digestion and absorption of nutrients in the broiler intestine, thus promoting ADG, and in agreement with this study, feeding fermented feeds can promote growth performance in poultry (30–32).

Meat quality is a critical factor in livestock production and is usually reflected by pH, meat color, freezing loss, drip loss, steaming loss, and amino acids (33). The pH level will directly affect the quality of the muscle, as the animal is slaughtered and its circulating nutrient supply is interrupted, causing a glycolytic reaction, which produces lactic acid, thus lowering the pH in the muscle (34). Consumers often base their decisions on the color of the meat, and the indications L^*^, a^*^, and b^*^ represent the color of the flesh. Higher L^*^ values indicate high muscle water exudation and easier formation of PSE meat. In general, the higher the a^*^ value and the lower the b^*^ value, the better the sensory quality of the meat (35). In the present research, we identified that FCMR supplementation had no significant effect on PH, meat color, drip loss, freezing loss, and amino acids of broilers and significantly reduced steaming loss, but data on PH and meat color of broilers showed better advantages, indicating that feeding FCMR could improve the sensory index of muscles.

The intestinal mucosa not only digests and absorbs nutrients but also serves as an essential defense barrier for the body (36). Better healthy digestion and absorption by the organism are indicated by longer gut villi and a shallower crypt depth (37, 38). This study found that FCMR supplementation increased VH and reduced CD in the small intestine of birds, possibly due to the intestinal benefits of the probiotics and bioactives in FCMR, which promote healthy intestinal growth. This suggests that FCMR can promote gut nutrient absorption and digestion in broiler chickens, thus promoting growth performance. Tight junctions are cellular gaps between adjacent epithelial cells, where claudin, occludin and ZO-1 comprise essential protein molecules in tight junctions (39). The tight junction proteins not only close the gaps among subepithelial tissues, creating a physical shield to prevent the passage of pro-inflammatory molecules, but also tight junction proteins are the main barrier to the paracellular pathway, preventing the passage of large molecules through the intestinal epithelium at the paracellular level while allowing the diffusion of ions and small molecules (40, 41). The IL family is a critical element in regulating inflammation, among which 1L-1β and 1L-6 plays a vital function in sustaining intestinal mucosal integrity and regulating intestinal immunity and are involved in many responses related to apoptosis or inflammation (42, 43). TNF-α is a classic indicator of inflammation and has been established as a critical modulator of the pro-inflammatory response (44). IFN-γ is associated with many infections, and elevated levels of IFN-γ can lead to automotive immune disease (45, 46). Chinese medicine residues contain many bioactive substances, such as polysaccharides, volatile oils, flavonoids, alkaloids and glycosides, which can promote the immunity of the body, and the bioactive substances in Chinese medicine can be improved by fermentation (47, 48). In our study, adding FCMR enhanced the relative mRNA expression of claudin, occluding, and ZO-1 and decreased the relative mRNA expression of inflammatory markers TNF-α, IFN-γ, 1L-1β, 1L-6 in broiler intestinal mucosa. Like our study, it was found that feeding herbs or fermented feeds can increase the integrity of the barrier in the intestine of poultry (49–52). Therefore, we believe that FCMR modulates gut mucosal barrier function and exhibits a strong anti-inflammatory effect, capable of reducing the number of inflammatory factors in animals.

As we all know, the intestinal microbiota plays a vital function in absorption and immune function, which helps to increase the growth performance of birds (53, 54). The intestinal microbiota regulates multiple host metabolic pathways, molding the immune response, preserving the gut mucosa integrity, and secreting certain enzymes and other metabolites (55, 56). Research has shown that adding fermented feeds to a diet can help regulate the makeup of intestinal microbes and prevent the gut from overreacting in an inflammatory way to infections, preserving intestinal health (57). Interestingly, our study found that supplementation with FCMR reduced ACE, Chao1, Shannon and Simpson indices of microorganisms in the broiler cecum, indicating a reduction in microbial diversity and abundance in the broiler cecum. This may be due to competition between dominant flora, which inhibits the value added of certain bacteria. Firmicutes and Bacteroides are the two major phyla in the poultry's intestines (58). Our study found that the most prevalent phylum in the cecum was *Bacteroides*. (66.32 vs. 68.70%) and *Firmicutes* was the second most abundant phylum. It indicates that feeding FCMR does not change the main bacterial species of the broiler cecum. Supplementation of FCMR significantly improved the abundance of *Spirochaetota* in the cecum of broiler chickens, which is an acid-producing bacterium that converts carbohydrates into simple volatile fatty acids(VFAS), thus providing energy to the body (59, 60). Supplementation of FCMR reduced the abundance of *Prevotellaceae_UCG-001, Desulfovibrio*, and *Fusobacterium* in broiler cecum, *Prevotellaceae_UCG-001* is more sensitive to inflammatory responses (61), and *Desulfurized* Vibrio can cause the host to reduce sulfites and sulfates received from the diet, as well as sulfated mucopolysaccharides, resulting in the production of the cellular toxic agent hydrogen sulfide (62). Studies have shown that patients suffering from fulminant colitis showed an elevated prevalence of *Desulfovibrio* and a significant inflammatory response, suggesting that FCMR reduced the frequency of sulfate-reducing *Desulfovibrio* and exerted its anti-inflammatory effects (63). Meanwhile, a total of 12 and 16 potential biomarkers in the CON and FCMR groups were significantly different, and the role of these biomarkers in broiler gut development needs to be further investigated. In conclusion, cecum microbiome mapping suggests that supplementation with FCMR may have the potential to increase colonization of beneficial microbes in the gut, minimize the number of bacteria that are responsible for dysfunction, which will lessen oxidative damage, maintain immunological control, and eventually preserve the integrity of the intestinal barrier.

Although our study found many benefits of FCMR supplementation for broilers, this was only at 5% supplementation and in the future, we will need to study the effects of different supplementation levels on broilers, which may help allow us to identify more advantageous supplementation levels than 5%. Combining the intestinal flora metabolome with the intestinal microbiota may allow us to better understand the mechanisms by which FCMR promotes intestinal health in broiler chickens. In addition, the applicability of FCMR needs to be verified by long-term feeding.

## 5. Conclusion

The present study showed that supplementation of FCMR in the diet can improve ADG and reduce FCR in broilers. Moreover, supplementation of FCMR can improve intestinal health by improving intestinal barrier function, intestinal inflammatory response and modifying microbial composition. In conclusion, we conclude that supplementation with 5% FCMR is beneficial to broiler health. The understanding of the regulatory role of FCMR on intestinal health and growth performance in broiler production warrants further study.

## Data availability statement

The datasets presented in this study can be found in online repositories. The name of the repository and accession numbers can be found at: NCBI; SRR23681981–SRR23681992.

## Ethics statement

The animal study was reviewed and approved by Leshan Academy of Agricultural Sciences.

## Author contributions

XHZ: conceptualization, methodology, formal analysis, investigation, and writing—original draft. SYL: conceptualization, methodology, and formal analysis. YLJ: formal analysis, data curation, and investigation. JCD and CPY: data curation and investigation. LJK: investigation. HDZ: investigation, supervision, and funding acquisition. XXC: investigation and supervision. All authors contributed to the article and approved the submitted version.
